# PREVALENCE OF GASTRIC CANCER AND PRENEOPLASTIC LESIONS: A CROSS-SECTIONAL STUDY IN A MEDIUM-RISK WESTERN POPULATION

**DOI:** 10.1590/S0004-2803.24612025-042

**Published:** 2025-10-27

**Authors:** Victor Cangussu Teixeira CAMPOS, Mayara Pezzini ARANTES, Luiz Roberto KOTZE, Leticia ROSEVICS, Susan Louise Kakitani TAKATA, Renata BRANDALISE, Eduardo Aimoré BONIN, Sandra TEIXEIRA

**Affiliations:** 1 Complexo Hospital de Clínicas da Universidade Federal do Paraná, Unidade de Endoscopia Digestiva, Curitiba, PR, Brasil.; 2 Complexo Hospital de Clínicas da Universidade Federal do Paraná, Unidade de Patologia, Curitiba, PR, Brasil.; 3 Complexo Hospital de Clínicas da Universidade Federal do Paraná, Unidade de Gastroenterologia, Curitiba, PR, Brasil.

**Keywords:** Gastric cancer, chronic atrophic gastritis, intestinal metaplasia, dysplasia, upper digestive endoscopy, Câncer gástrico, gastrite atrófica crônica, metaplasia intestinal, displasia, endoscopia digestiva alta

## Abstract

**Background:**

The late diagnosis of gastric cancer, which is usually diagnosed via upper digestive endoscopy, may be attributed to the failure to detect precursor lesions. This study aimed to determine the prevalence of gastric cancer and its precursor lesions in individuals undergoing routine upper gastrointestinal endoscopies at a tertiary hospital in Brazil.

**Methods:**

Patients aged >40 years who had undergone diagnostic endoscopic examinations in 2017 at our institution were included in this retrospective cross-sectional study. We exclude patients with more than one examination in the time-period, without gastric biopsies, and those with autoimmune atrophic gastritis and history of gastric surgery. The histopathological findings were reviewed by two gastrointestinal pathologists. The Operative Link on Gastritis Assessment (OLGA) classification was used in cases wherein gastric mapping was performed. Descriptive statistical analysis for the diagnostic findings of the malignant precursor lesions and gastric cancer was performed using Chi-square test. Statistical significance was set at *P*<0.05.

**Results:**

Among the 1,071 patients (64.6% females; age 60±10.4 years old) who underwent endoscopic examinations, 277 (25.9%) were diagnosed with malignant precursor lesions or cancer, three (0.3%) dysplastic lesions, and 12 (1.1%) neoplasms. A total of 888 patients underwent gastric mapping; OLGA III and IV stages were observed in 46 (5.2%) patients. Chi-square test revealed significant correlations between dysplastic and neoplastic lesions and male sex, age >80 years and the prevalence of chronic atrophic gastritis and intestinal metaplasia, and age 40-49 years and the prevalence of Helicobacter pylori infection.

**Conclusion:**

Precursor lesions for gastric cancer were observed in up to 25% of the patients, with a predominance of low-risk lesions. Further prospective studies must be conducted to evaluate the risk of gastric cancer in individuals with precursor lesions and formulate prevention strategies.

## HIGHLIGHTS

•Due to the intermediate prevalence of gastric cancer in Brazil, this study aimed to determine the prevalence of gastric cancer and especially its precursor lesions in individuals undergoing routine upper gastrointestinal endoscopies at a tertiary hospital in Brazil.

•This study evaluated diagnostic upper endoscopies in patients older than 40 years old during a year period, using the operative link on gastritis assessment (OLGA) classification by a pathologist specialized in gastrointestinal pathology.

•The studied had found correlations between dysplastic and neoplastic lesions and male sex, age >80 years and the prevalence of chronic atrophic gastritis and intestinal metaplasia, and age 40-49 years and the prevalence of Helicobacter pylori infection.

•The group of patients that were suggested to be at higher risk of dysplastic lesions need further prospective studies to formulate prevention strategies.

## RESUMO

**Contexto:**

O diagnóstico tardio do câncer gástrico, que geralmente é diagnosticado por endoscopia digestiva alta, pode ser atribuído à falha de diagnóstico de lesões precursoras. Este estudo teve como objetivo determinar a prevalência de câncer gástrico e suas lesões precursoras em indivíduos submetidos a endoscopias gastrointestinais altas de rotina em um hospital terciário no Brasil.

**Métodos:**

Pacientes com idade >40 anos que foram submetidos a exames endoscópicos diagnósticos em 2017 em nossa instituição foram incluídos neste estudo transversal retrospectivo. Pacientes que foram submetidos a exames endoscópicos de acompanhamento, pacientes que não foram submetidos a biópsias gástricas e aqueles com gastrite atrófica autoimune e histórico de cirurgia gástrica foram excluídos. Os achados histopatológicos foram revisados por dois patologistas gastrointestinais. A classificação Operative Link on Gastritis Assessment (OLGA) foi usada nos casos em que o mapeamento gástrico foi realizado. A análise estatística descritiva para os achados diagnósticos das lesões precursoras malignas e câncer gástrico foi realizada usando o teste qui-quadrado. A significância estatística foi definida em *P*<0,05.

**Resultados:**

Entre os 1.071 pacientes (64,6% mulheres; idade 60 ± 10,4 anos) que foram submetidos a exames endoscópicos, 277 (25,9%), três (0,3%) e 12 (1,1%) pacientes foram diagnosticados com lesões precursoras malignas ou câncer, lesões displásicas e neoplasias, respectivamente. Um total de 888 pacientes foram submetidos a mapeamento gástrico; os estágios OLGA III e IV foram observados em 46 (5,2%) pacientes. O teste qui-quadrado revelou correlações significativas entre lesões displásicas e neoplásicas e sexo masculino, idade >80 anos e a prevalência de gastrite atrófica crônica e metaplasia intestinal, e idade 40-49 anos e a prevalência de infecção por *Helicobacter pylori*.

**Conclusão:**

Lesões precursoras de câncer gástrico foram observadas em até 25% dos pacientes, com predominância de lesões de baixo risco. Mais estudos prospectivos devem ser conduzidos para avaliar o risco de câncer gástrico em indivíduos com lesões precursoras e formular estratégias de prevenção.

## INTRODUCTION

Adenocarcinoma, the most prevalent type of gastric neoplasm, accounts for approximately 90% of all cases of gastric cancer (GC)^1^. First described by Pelayo-Correa in 1988^2^, the carcinogenesis of GC progresses sequentially. Repeated injury to the normal mucosa leads to chronic gastritis, which subsequently evolves into chronic atrophic gastritis (CAG), intestinal metaplasia (IM), dysplasia, and carcinoma. Chronic *Helicobacter Pylori* (HP) infection, classified as a group 1 carcinogen for GC by the World Health Organization since 1994^3^, has been identified as the main etiological agent activating this cascade. Interaction between several factors related to the bacteria, host, and environment results in HP-induced chronic inflammation and subsequent carcinogenesis.

The presence of CAG and IM have been linked with the risk of developing GC. For instance, the risk of developing GC is low in cases wherein CAG and IM are restricted to the antrum, whereas it is high when these changes extend to the gastric body or are present outside the cardia^4^ ^,^ ^5^. Other characteristics, such as a family history of GC, residing in areas with a high prevalence of GC, ethnicity^6^, histological findings of dysplasia, and stages III and IV of the Operative Link on Gastritis Assessment (OLGA) or Operative Link on Gastric Intestinal Metaplasia Assessment system^5^, increase the risk of cancer. Therefore, Endoscopic Societies worldwide recommend screening high-risk patients and establishing criteria for follow-up based on these characteristics^5^ ^,^ ^7^ ^,^ ^8^.

GC, one of the five malignant tumors with the highest incidence in Brazil, is associated with a high mortality rate^9^. GC is the fifth most commonly occurring type of cancer across the globe. Moreover, according to Global Burden of Disease data from the Institute for Health Metrics and Evaluation and the Global Cancer Observatory report^10^, it ranks fourth in terms of mortality, accounting for approximately 769.000 deaths. The high mortality rate for GC may be attributed to its late diagnosis, as only 5-20% of cases are diagnosed early in Brazil and most Western countries. Increasing the detection rate of preneoplastic lesions, such as CAG and IM, and identifying patients at high risk of developing GC who require adequate follow-up could facilitate the early diagnosis of GC. However, few studies have evaluated the prevalence of preneoplastic gastric lesions in Brazil despite the available evidence. Moreover, the systematization of biopsies has not been performed, which hinders the formulation of preventive measures, such as screening, that can be performed routinely for colorectal cancer^9^ ^,^ ^11^ ^-^ ^16^. Therefore, this study aimed to assess the prevalence of GC and its precursor lesions at a tertiary Digestive Endoscopy Unit (DEU) in Southern Brazil.

## METHODS

The data extracted from the electronic records of the DEU and the archives of the Pathology Unit of the Clinics Hospital Complex of the Federal University of Paraná (CHC-UFPR), a tertiary public hospital in Southern Brazil, were used in this retrospective cross-sectional study. A total of 2.000-5.000 examinations are conducted annually at the DEU.

Convenience sampling was performed by reviewing the reports of the upper gastrointestinal endoscopy (UGIE) conducted at the DEU/CHC-UFPR in 2017. All endoscopic examinations were performed by a trainee endoscopist using the gastroscopes Olympus® GIF-Q150 (9.8 mm diameter) and Fujinon® EG-530WR (9.3 mm diameter) with white light under the supervision of an experienced endoscopist. Chromoendoscopy with indigo carmine was used only in cases with lesions that were suspected to be malignant. Patients aged >40 years who were scheduled to undergo diagnostic UGIE were included in this study. The exclusion criteria were as follows: absence of gastric biopsies, autoimmune atrophic gastritis, history of gastric surgery, emergency examinations (owing to upper gastrointestinal bleeding, nasoenteral tube positioning, and drop in hemoglobin count without externalization), inadequate preparation, and examinations conducted for therapeutic purposes or follow-up of gastric lesions. The most recent examination was selected if the patients had undergone more than one UGIE in 2017.

The patients were classified according to age, sex, presence of HP infection, and the endoscopic and histopathological findings of the lesions suspected to be preneoplastic lesions (CAG, IM, or dysplasia) and neoplasia^5^ ^,^ ^17^. Patients who had undergone individualized biopsies (properly identified in biopsy vials) of the gastric antrum and body were included in the gastric mapping subgroup. The OLGA system^5^ ^,^ ^17^ was used to determine the extent of preneoplastic lesions in this subgroup. All patients had at least four biopsies’ samples, two in the gastric antrum and two in the gastric body, but some patients had also biopsies from the incisura angularis.

The biopsies performed after assessing the presence of endoscopic findings were reviewed by two pathologists specializing in gastrointestinal diseases in the case of patients with suspected precursor lesions and GC. Biopsies were classified based on the presence or absence of CAG, IM (complete and incomplete), dysplasia (low- or high-grade), GC and HP (Giemsa staining). The GC stage was classified as early or advanced.

The ages of the patients are presented as mean, median, minimum and maximum values, and standard deviation. Categorical variables are presented as frequencies and percentages. The chi-squared test was used to analyze the association between two categorical variables. Statistical significance was set at *P*<0.05. All statistical analyses were performed using Stata/SE software, version 14.1 (StataCorp LLC, College Station, Texas, USA).

This study was approved by the local Ethical Committee (18583119.5.0000.0096).

## RESULTS

Among the 2.243 consecutive patients who underwent endoscopic examinations for various indications, 1.112 patients were included after the application of the exclusion criteria (Table 1). In addition, patients with histopathological specimens that could not be analyzed owing to poor quality were also excluded from the analysis. Thus, 1.071 patients aged 40-97 years (mean age: 60 years, 64.6% females) were included in the final analysis. More than half of the patients were aged 50-70 years (63.1%). Table 2 presents the demographic findings.

**TABLE 1:** Total excluded examinations and reason for exclusion of individuals who underwent consecutive endoscopic examinations.

Grounds for exclusion	Number of examinations	%
Without biopsy	613	54.2%
Therapeutic endoscopies	182	16.1%
Gastrectomy	152	13.4%
Emergency examinations	82	7.2%
Other	63	5.6%
Material lost or not analyzable	39	3.5%
Total	1,131	100.0%

**TABLE 2:** Age and sex of the consecutive individuals with histopathological alterations who underwent endoscopic examinations (n=1,071).

Variable	Classification	Result*
Age (years)		60±10.4 (40-97)
Age group	40-49	187 (17.5)
	50-59	348 (32.5)
	60-69	328 (30.6)
	70-79	173 (16.2)
	≥80	35 (3.3)
Sex	Female	692 (64.6)
	Male	379 (35.4)

*Mean ± standard deviation (percent).

**FIGURE 1:**
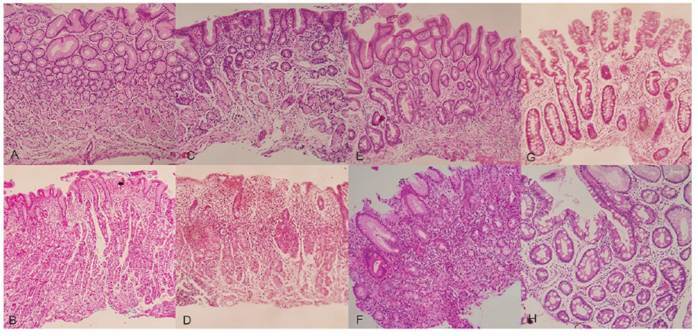
Gastric atrophy grading. A - normal antral mucosa, B - normal oxyntic mucosa. C, D - mild, non metaplastic atrophy of antrum (C) and corpus (D). Glands are diminished in number and lamina propria is more visible. Cases with up to 30% atrophy (mean for all fragments) were classified as mild. E, F - moderate atrophy of antrum (E) and corpus (F) with intestinal metaplasia. There is substitution of normal antral and oxyntic glands by intestinal type epithelium with goblet cells. In the corpus, there is also pseudopyloric metaplasia. Cases with 30-60% atrophy (mean for all fragments) were classified as moderate. G, H - severe atrophy of antrum (G) and corpus (H), with intestinal metaplasia. The mucosa is almost entirely atrophic and metaplastic. Cases with >60% atrophy were classified as severe atrophy.

In total, 888 out of the 1.071 patients underwent histological mapping (histological differentiation of material obtained from the gastric body and antrum), thereby facilitating the determination of the extent of preneoplastic lesions and the application of the OLGA system^17^. Figure 1 shows the histological grading of gastric atrophy. Advanced OLGA stages III or IV were observed in 5.2% of patients, whereas OLGA stages I or II were observed in 20.6%.

CAG and IM were observed in 25.9% and 20.9% of the patients, respectively. Low-grade dysplastic lesions were observed in three patients. Twelve patients were diagnosed with GC, among whom two (16.6%) had early-stage GC. Table 3 presents the prevalence of all alterations identified in the present study.

**TABLE 3:** Histopathological alterations observed in the individuals who underwent endoscopic examinations (n=1,071).

Variable	n (%)
Chronic atrophic gastritis	277 (25.9)
Intestinal metaplasia	224 (20.9)
Complete intestinal metaplasia	123 (11.5)
Incomplete intestinal metaplasia	109 (10.2)
Dysplastic and neoplastic lesions	
Low-grade dysplasia	3 (0.3)
High-grade dysplasia	0 (0)
Early gastric cancer	2 (0.2)
Advanced gastric cancer	10 (0.9)
*Helicobacter pylori* infection	355 (33.1)

Tables 4 AND 5 present the results of the statistical analyses performed to assess differences between the groups in terms of sex, age, and presence of HP infection. The chi-squared test revealed a significantly higher prevalence of dysplastic and neoplastic lesions in male patients. Similarly, the prevalence of CAG and IM was higher in patients aged >80 years, and the prevalence of HP infection was higher in patients aged 40-49 years.

**TABLE 4:** Association between age and preneoplastic and neoplastic gastric lesions with histopathological alterations in individuals who underwent endoscopic examinations (n=1,071).

Variable	Age (years)					P*
	40-49 (n=187)	50-59 (n=348)	60-69 (n=328)	70-79 (n=173)	≥80 (n=35)	
Overall result	36 (19.3)	81 (23.3)	85 (25.9)	65 (37.6)	18 (51.4)	<0.001
Chronic atrophic gastritis	34 (18.2)	77 (22.1)	84 (25.6)	64 (37)	18 (51.4)	<0.001
Intestinal metaplasia	27 (14.4)	62 (17.8)	68 (20.7)	53 (30.6)	14 (40)	<0.001
Complete intestinal metaplasia	16 (8.6)	37 (10.6)	34 (10.4)	28 (16.2)	8 (22.9)	0.034
Incomplete intestinal metaplasia	11 (5.9)	27 (7.8)	37 (11.3)	27 (15.6)	7 (20)	0.003
*Helicobacter pylori* infection	80 (42.8)	122 (35.1)	107 (32.6)	35 (20.2)	11 (31.4)	<0.001
Dysplastic and neoplastic lesions	2 (1.1)	4 (1.1)	5 (1.5)	3 (1.7)	1 (2.9)	-

Chi-square test, *P*<0.05. (-) Test not applicable because of the low frequency of these combinations. Note: Findings of intestinal metaplasia, classified as complete or incomplete, may overlap. For example, an individual may present both complete and incomplete forms.

**TABLE 5:** Gender and alterations in Gastrointestinal Upper Endoscopies (n=888).

Variable	Sex		P*
	Female (n=579)	Male (n=309)	
Overall result	166 (24)	119 (31.4)	0.009
Chronic atrophic gastritis	166 (24)	111 (29.3)	0.058
Intestinal metaplasia	133 (19.2)	91 (24)	0.065
Complete intestinal metaplasia	75 (10.8)	48 (12.7)	0.370
Incomplete intestinal metaplasia	64 (9.2)	45 (11.9)	0.174
OLGA			
0	442 (76.3)	217 (70.2)	
I/II	109 (18.8)	74 (23.9)	
III/IV	28 (4.8)	18 (5.8)	0.138
*H. pylori* presence	233 (33.7)	122 (32.2)	0.623
Dysplastic and neoplastic lesions	1 (0.1)	14 (3.7)	<0.001

*Chi-square test, *P*<0.05. (-) Test not applicable because of the low frequency of these combinations.

## DISCUSSION

Precursor lesions of GC have garnered an increasing amount of interest as the prevention of these lesions increases the odds of early diagnosis and treatment^5^ ^-^ ^8^. The early diagnosis of GC has a positive effect on public health as it decreases mortality and preserves the resources necessary for the treatment of neoplasms in advanced stages^18^. A total of 1.071 patients (64.6% females, average age: 60±10.4 years) underwent endoscopic examinations in the present study. Malignant precursor lesions or cancer were detected in 25.9% of these cases. Gastric mapping performed according to the ESGE guidelines revealed OLGA stages III and IV in 5.2% of patients. Furthermore, the chi-squared test revealed statistically significant correlations between male sex and the presence of dysplastic and neoplastic lesions and between age >80 years and the presence of CAG and IM.

The prevalence rates of CAG and IM were 25.9% and 20.9%, respectively, in the present study. However, the global prevalence rates of CAG and IM are 16.2% and 13.2%, respectively. Thus, the prevalence rates of these lesions vary based on the incidence of GC in each country, as they are proportionally higher in places with a higher GC incidence. The prevalence rates of CAG and IM were 26.7% and 16.2%, respectively, in regions with a high incidence of GC. In contrast, the prevalence rates of CAG and IM were 7.3% and 7.7%, respectively, in regions with a low or moderate incidence of GC^19^. Previous studies conducted in Brazil have reported prevalence rates of 3% and 15% for CAG and IM, respectively^16^. Therefore, a separate analysis must be conducted for each state to properly assess the risk, considering the proportion of the country and differences in access to healthcare across Brazil.

The first study with the largest sample size on the prevalence of these lesions was conducted in 2015 in Sweden, a country with a low incidence of GC^20^. A total of 405.172 patients who had undergone UGIE with gastric biopsies between 1979 and 2011 were followed up in this study^20^. The prevalence rate of CAG was 4.2%^20^. Another study conducted in the USA in 2015 that included 895.323 participants revealed that the prevalence rate of CAG was 12.8%. Few studies have investigated the prevalence of preneoplastic gastric lesions in South America^14^ ^-^ ^16^ ^,^ ^21^ ^,^ ^22^. A study conducted in Venezuela in 2007 that included 2.200 participants revealed that the prevalence rate of CAG was 15%. The prevalence rate of CAG in Brazil has been reported to be moderate^21^; however, previous studies conducted in Brazilian states other than Paraná have reported that the prevalence rate of CAG ranges from 16.7-30.18%^14^ ^-^ ^16^ ^,^ ^22^. The present study is the first to investigate the prevalence of these lesions in the State of Paraná, Brazil.

Only three patients (0.3%) had low-grade dysplastic lesions in the present study. This finding is consistent with the findings of most previous studies, which have reported prevalence rates of <1% (for instance, the Swedish study [0.6%] cited above)^20^. The prevalence rates observed in the present study were lower than those reported in the study conducted by Plummer in 2007 in Venezuela (6%)^23^. However, unlike the present study, which was a retrospective study that analyzed consecutive UGIEs conducted at the DEU/CHC-UFPR, the study conducted by Plummer was prospective, which may have influenced the endoscopic analysis.

Twelve cases of GC were identified in the present study, among which 16.6% were detected early. On average, GC is detected during early stages in 5-20% of cases in the Western world^24^ ^-^ ^26^. Although only two cases were identified in the present study, this was the highest rate (16.6%) reported among studies conducted in Brazil (0-15.8%)^11^ ^-^ ^13^ ^,^ ^22^ ^,^ ^27^.

The present study demonstrated that the prevalence of preneoplastic lesions (GCA and IM) increases significantly with age and that the presence of HP was associated with more advanced OLGA stages (III and IV). Hence, older age is an important risk factor for the development of preneoplastic lesions. The time of contracting HP infection may also have played an important role, as most infections occur during childhood^21^.

The stage of CAG was classified according to the OLGA system in cases wherein the site from which the gastric sample was obtained (body or antrum) could be identified. Although the total percentage of patients with advanced OLGA stages was only 5.2%, a higher probability of the incidence of IM in patients with the most advanced stages of CAG was demonstrated, reinforcing the validity of the Pelayo-Correa carcinogenic cascade^2^. The low prevalence of advanced OLGA stages may be attributed to the quality of gastric sampling. The sensitivity of the protocol for more advanced stages can be increased by obtaining a greater number of fragments than usual.

The OLGA system stratifies patients into categories at high and low risk of developing GC, thereby enabling the initiation of endoscopic follow-up (GC surveillance) for those at higher risk. However, the OLGA system is considered a less sensitive strategy for the risk stratification of GC. The most recent ESGE guidelines^5^, which have made broader recommendations, propose follow-up for a larger group of patients, including those with non-extensive IM, with the following characteristics: incomplete type, first-degree relatives with GC, history of autoimmune gastritis, and persistent HP infection. Thus, the number of patients indicated to undergo endoscopic follow-up will increase if the criteria proposed by the ESGE are considered.

The number of patients indicated to undergo follow-up based on the ESGE guidelines and OLGA system (only stages III and IV) were compared in the present study. In the subsample of patients who underwent OLGA staging (n=888), stages 0, I, and II (low-risk for GC) accounted for 842 cases, whereas stages III and IV (high-risk for GC) accounted for 46 cases. However, 65 patients in the low-risk group who were not indicated to undergo follow-up according to the OLGA system had incomplete IM. The number of patients indicated to undergo follow-up increased from 46 to 111, indicating a 2.4 - fold increase when only one of the criteria proposed by the ESGE for non-extensive IM was considered.

The latest consensus on HP infection recommends follow-up only for patients with OLGA stages III and IV^21^. This is the only Brazilian guideline recommending the follow-up of preneoplastic gastric lesions at present. The adoption of broader criteria for GC risk stratification could increase the number of patients under surveillance, thereby increasing the early diagnosis of GC.

A slight tendency toward a higher prevalence rate of preneoplastic lesions was observed in males. Similarly, dysplastic and neoplastic lesions were observed significantly more frequently in males. Similar findings have been reported in males in a 2014 study that conducted a systematic review and meta-analysis of studies on the prevalence of GC and its preneoplastic lesions published between 1980 and 2012^19^. The present study revealed a statistically significant correlation between male sex and the higher incidence of dysplastic precursor lesions, which is similar to the findings of a previous Brazilian study conducted in Piauí that was concordant with the national gastric cancer rates^9^ ^,^ ^12^. However, this finding differs from those of other studies conducted in Brazil^13^ ^,^ ^15^.

The present study was limited by its retrospective and single-center design. Although the sample size was small, the preset study provided real-life data over a year via a high-volume endoscopy service. Another limitation was the limited number of chromoscopies performed and the lack of magnification; however, this study represents how most endoscopic examinations are performed clinically in Brazil. Furthermore, two endoscopists and two pathologists performed the study.

The present study demonstrated a high prevalence of malignant gastric precursor lesions among patients undergoing endoscopic examinations at a tertiary hospital. In addition, a predominance of lesions with a low risk of progression to GC was also observed. The prevalence of advanced malignant lesions was higher among the patients diagnosed with GC. These data suggest that screening and follow-up of gastric precursor lesions may positively impact public health, thereby highlighting the requirement for implementing preventive measures. Increasing the number of early diagnoses could help reduce costs and improve the quality of life of the general population. This study raises questions regarding the requirement for systematization of tests and biopsies among patients undergoing upper digestive endoscopies in Brazil for the screening and follow-up of gastric lesions that are precursors of neoplasia.

## References

[1] Lordick F, Carneiro F, Cascinu S, Fleitas T, Haustermans K, Piessen G (2022). Gastric cancer: ESMO Clinical Practice Guideline for diagnosis, treatment and follow-up. Ann Oncol.

[2] Correa P (1988). Perspectives in cancer research a human model of gastric carcinogenesis. Nutrition.

[3] Wen S, Moss SF (2009). Helicobacter pylori virulence factors in gastric carcinogenesis. Cancer letters.

[4] Marcos P, Brito-Gonçalves G, Libânio D, Pita I, Castro R, Sá I (2020). Endoscopic grading of gastric intestinal metaplasia on risk assessment for early gastric neoplasia: can we replace histology assessment also in the West?. Gut.

[5] Pimentel-Nunes P, Libânio D, Marcos-Pinto R, Areia M, Leja M, Esposito G (2019). Management of epithelial precancerous conditions and lesions in the stomach (MAPS II): European Society of Gastrointestinal Endoscopy (ESGE), European Helicobacter and Microbiota Study Group (EHMSG), European Society of Pathology (ESP), and Sociedade Portuguesa de Endoscopia Digestiva (SPED) guideline update 2019. Endoscopy.

[6] Altayar O, Davitkov P, Shah SC, Gawron AJ, Morgan DR, Turner K (2020). AGA technical review on gastric intestinal metaplasia-epidemiology and risk factors. Gastroenterology.

[7] Evans JA, Chandrasekhara V, Chathadi KV, Decker GA, Early DS, ASGE Standards of Practice Committee (2015). The role of endoscopy in the management of premalignant and malignant conditions of the stomach. Gastrointestinal endoscopy.

[8] Banks M, Graham D, Jansen M, Gotoda T, Coda S, Di Pietro M (2019). British Society of Gastroenterology guidelines on the diagnosis and management of patients at risk of gastric adenocarcinoma. Gut.

[9] Instituto Nacional de Câncer (Brasil) (2022). Estimativa 2023: incidência de câncer no Brasil / Instituto Nacional de Câncer.

[10] Sung H, Ferlay J, Siegel RL, Laversanne M, Soerjomataram I, Jemal A, Bray F (2024). Global cancer statistics 2022: GLOBOCAN estimates of incidence and mortality worldwide for 36 cancers in 185 countries. CA Cancer J Clin.

[11] Pinto CE (2001). Early gastric cancer: Review of 47 cases from the national cancer institute in the last five years. Rev Col Bras Cir.

[12] Campelo JCL, Lima LC (2012). Perfil Clinicoepidemiológico do Câncer Gástrico Precoce em um Hospital de Referência em Teresina, Piauí. Rev bras cancerol.

[13] Braga-Neto MB, Carneiro JG, de Castro Barbosa AM, Silva IS, Maia DC, Maciel FS (2018). Clinical characteristics of distal gastric cancer in young adults from Northeastern Brazil. BMC cancer.

[14] Rodrigues MF, Guerra MR, Alvarenga AVR, Souza DZO, Costa RAVES, Cupolilo SMN (2019). Helicobacter pylori infection and gastric cancer precursor lesions: prevalence and associated factors in a reference laboratory in southeastern Brazil. Arquivos de gastroenterologia.

[15] Motta CR, Cunha MP, Queiroz DM, Cruz FW, Guerra EJ, Mota RM (2008). Gastric precancerous lesions and Helicobacter pylori infection in relatives of gastric cancer patients from northeastern Brazil. Digestion.

[16] Muller LB, Fagundes RB, Moraes CC, Rampazzo A (2007). Prevalence of Helicobacter pylori infection and gastric cancer precursor lesions in patients with dyspepsia. Arq Gastroenterol.

[17] Rugge M, Correa P, Di Mario F, El-Omar E, Fiocca R, Geboes K (2008). OLGA staging for gastritis: A tutorial. Dig Liver Dis.

[18] Areia M, Carvalho R, Cadime AT, Rocha Gonçalves F, Dinis-Ribeiro M (2013). Screening for gastric cancer and surveillance of premalignant lesions: a systematic review of cost-effectiveness studies. Helicobacter.

[19] Marques-Silva L, Areia M, Elvas L, Dinis-Ribeiro M (2014). Prevalence of gastric precancerous conditions: a systematic review and meta-analysis. Eur J Gastroenterol Hepatol.

[20] Song H, Ekheden IG, Zheng Z, Ericsson J, Nyrén O, Ye W (2015). Incidence of gastric cancer among patients with gastric precancerous lesions: observational cohort study in a low risk Western population. BMJ.

[21] Coelho LGV, Marinho JR, Genta R, Ribeiro LT, Passos MDCF, Zaterka S (2018). IVTH BRAZILIAN CONSENSUS CONFERENCE ON HELICOBACTER PYLORI INFECTION. Arq Gastroenterol.

[22] Dietz J, Ulbrich-Kulcynski JM, Souto KE, Meinhardt NG (2012). Prevalence of upper digestive endoscopy and gastric histopathology findings in morbidly obese patients. Arq Gastroenterol.

[23] Plummer M, van Doorn LJ, Franceschi S, Kleter B, Canzian F, Vivas J (2007). Helicobacter pylori cytotoxin-associated genotype and gastric precancerous lesions. J Natl Cancer Inst Monogr.

[24] Everett SM, Axon AT (1997). Early gastric cancer in Europe. Gut.

[25] Clark CJ, Thirlby RC, Picozzi V, Schembre DB, Cummings FP, Lin E (2006). Current problems in surgery: Gastric cancer. Curr Probl Surg.

[26] Desai AM, Pareek M, Nightingale PG, Fielding JW (2004). Improving outcomes in gastric cancer over 20 years. Gastric Cancer.

[27] Gama-rodrigues J, Pinotti HW (1994). Tratado de Clínica Cirúrgica do Aparelho Digestivo.

